# RECENT ADVANCES IN PSORIASIS: BENCH TO BEDSIDE

**DOI:** 10.4103/0019-5154.62749

**Published:** 2010

**Authors:** Siba P Raychaudhury

**Affiliations:** *From Division of Allergy and Clinical Immunology, University of California Davis, School of Medicine and VA Sacramento Medical Centre, CA 95618, USA.*

Psoriasis is likely to be the most common autoimmune disease. Nearly 2% of the global population suffers from psoriasis. This is a nonfatal, life-long disease, but on occasions, psoriasis can be a source of significant morbidity. Extensive pustular lesions generalized involvement of the body (erythroderma), and psoriatic arthritis are severe complications of psoriasis. In the last two decades, rapid progress has been made in understandings of the cellular and molecular pathways of inflammation that contribute to the disease pathogenesis. Psoriasis is characterized by hyperplasia of epidermal keratinocytes, angiogenesis, and infiltration of T-lymphocytes, neutrophils, and other types of leukocyte in the affected skin. The proliferative keratinocyte response is thought to be due to activation of the cellular immune system, with T-cells, dendritic cells, and various immune-related cytokines and chemokines implicated in pathogenesis. In a relatively short period, psoriasis has been conceptualized as a T-lymphocyte-mediated autoimmune disease, and new biological therapies that target T-cells have just entered routine clinical practice. However, current observations suggest that although activated T-lymphocytes have an undisputed role in the pathogenesis of psoriasis, there are other regulatory systems that contribute to the inflammatory and proliferative processes of psoriasis. A complete understanding of the pathogenesis of psoriasis and psoriatic arthritis is lacking. Cytokines, chemokines, adhesion molecules, growth factors like NGF, neuropeptides, and T-cell receptors all act in an integrated way to evolve into unique inflammatory and proliferative processes typical for psoriasis.

In this issue, we are publishing invited articles from the leading academic institutes who are carrying out cutting edge clinical and bench research to solve the puzzles of psoriatic disease. These articles will cover latest finding of genetic studies, animal models and inflammatory cascades in psoriasis. In addition these articles will provide updates on the biologics for psoriasis with a special focus on the IL-23/Th-17/IL-17 immune axis in psoriatic disease.

